# Investigation of the most common clinical and imaging findings and the role of tubulin genes in the etiology of malformations of cortical development

**DOI:** 10.3906/sag-1901-170

**Published:** 2020-10-22

**Authors:** Özge AKSEL KILIÇARSLAN, Esra ATAMAN, Semra GÜRSOY, Gürkan GÜRBÜZ, Aycan ÜNALP, Pınar GENÇPINAR, Nihal OLGAÇ DÜNDAR, Selvinaz EDİZER, Ayfer ÜLGENALP, Özlem GİRAY BOZKAYA

**Affiliations:** 1 Department of Medical Genetics, Faculty of Medicine, Dokuz Eylül University, İzmir Turkey; 2 Department of Pediatric Genetics, Faculty of Medicine, Dokuz Eylül University, İzmir Turkey; 3 Department of Pediatric Neurology, Dr. Behcet Uz Child Disease and Pediatric Surgery Training and Research Hospital, İzmir Turkey; 4 Department of Pediatric Neurology, Tepecik Training and Research Hospital, İzmir Turkey

**Keywords:** Cortical dysplasia, tubulinopathies, *TUBA1A*, *TUBB2B*, *TUBB3*

## Abstract

**Background/aim:**

The number of reports on the role of tubulin gene mutations ( *TUBA1A, TUBB2B,* and *TUBB3* ) in etiology of malformations of cortical development has peaked in recent years. We aimed to determine tubulin gene defects on a patient population with simple and complex malformations of cortical development, and investigate the relationship between tubulin gene mutations and disease phenotype.

**Materials and methods:**

We evaluated 47 patients with simple or complex malformations of cortical development, as determined by radiological examination, for demographic features, clinical findings and mutations on *TUBA1A, TUBB2B,* and *TUBB3* genes.

**Results:**

According to the magnetic resonance imaging findings, 19 patients (40.5%) had simple malformations of cortical development and 28 (59.5%) patients had complex malformations of cortical development. Focal cortical dysplasia was the most common simple malformation, lissencephaly was the most common coexisting cortical malformation, and corpus callosum anomalies were the most common coexisting extracortical neurodevelopmental abnormalities. None of the patients had genetic alterations on *TUBA1A, TUBB2B,* and *TUBB3* genes causing protein dysfunction. On the other hand, the frequencies of some polymorphisms were higher when compared to the literature.

**Conclusion:**

It is crucial to identify the etiology in patients with malformations of cortical development in order to provide appropriate genetic counseling and prenatal diagnosis. We consider that multicenter studies with higher patient numbers and also including other malformations of cortical development-related genes are required to determine underlying etiological factors of malformations of cortical development patients.

## 1. Introduction

Malformations of cortical development (MCD) are an important cause of refractory epilepsy, intellectual disability, developmental delay, and neurological deficits. Studies in recent years have shown that mutations on genes encoding i) different tubulin isotypes ( *TUBA1A* , *TUBA8* , *TUBB2A* , *TUBB2B* , *TUBB3* , *TUBB* , *TUBG1* ), ii) microtubule-associated proteins ( *LIS1* , *DCX* , *KIF2A* , *KIF5C* ), or iii) microtubule-based motor proteins (kinesin, dynein) play a role in etiology of MCD [1].

Tubulinopathies include a broad spectrum of brain malformations (microlissencephaly, classical lissencephaly, polymicrogyria, schizencephaly, corpus callosum agenesia or hypoplasia, dysmorphic basal ganglia, brain stem and cerebellar hypoplasia, ventriculomegaly, and vermis hypoplasia) and clinical findings (cognitive and/or motor impairments, epilepsy, congenital microcephaly). Furthermore, congenital fibrosis of the extraocular muscles and progressive sensorimotor polyneuropathy could be seen in specific *TUBB3* and *TUBB2* mutations. Tubulinopathies generally are inherited in an autosomal dominant manner. On the other hand, some reported tubulinopathy families with *TUBA8* mutations had autosomal recessive inheritance https://www.ncbi.nlm.nih.gov/books/NBK350554/ [2,6].

In this study, we aimed to determine *TUBA1A* , *TUBB2B* , and *TUBB3* gene defects in simple MCD and complex MCD (with additional cortical and/or extracortical neurodevelopmental malformations) patient group with unknown etiology, and explore relationship between disease phenotype and clinical progression in patients with tubulin gene mutations.

## 2. Materials and methods

### 2.1. Patients

Patients referred to our department from three pediatric hospitals for radiologically diagnosed MCDs with unidentified etiology for a 6-month period were included in this study. All participants were below 18 years of age. Detailed information was given to all participants and their relatives. Written informed consents were obtained from the all participants and/or their parents. The study protocol received approval by Dokuz Eylül University Non-Invasive Researches Ethical Review Board and the approval code is 2015/12-51.

### 2.2. Data collection

Information obtained from examinations and medical records were recorded in data collection forms. All patients were examined by a pediatric neurologist and clinical geneticist. The following information were recorded; personal information, prenatal history, history of epilepsy (age of onset, type of seizures, EEG findings, response to antiepileptics), motor development stages, head circumference, examination notes, imaging results, hearing test results, genetic test results, and pedigree. Cognitive function and motor development were evaluated using the Wechsler Intelligence Scale of Children-Revised (WISC-R) and the Denver Developmental Screening Test, respectively. All MR images were evaluated by two pediatric neuroradiologists. The classification of the MCD subtype was made according to the Barkovich classification system. MCD associated with additional cortical or extracortical malformations were classified as complex MCD.

### 2.3. Mutation screening

For DNA isolation, 5 mL of peripheral blood sample was collected. DNA was extracted from lymphocytes using standard methods (High Pure PCR Template Preparation Kit, Roche™). All coding exons and exon-intron junctions of *TUBA1A* , *TUBB2B* , and *TUBB3* genes were amplified with polymerase chain reaction (PCR) using Helix Amp™ Hot-Taq Polymerase [Ver 2.0] (with dNTP Mix) (NanoHelix) for *TUBA1A* gene part 4b and *TUBB3* gene part 4a, and Helix Amp™ Ready-2X Multiplex version 2.0 PCR mix (NanoHelix) for the rest of the other parts. Protocols were performed according to the standards provided by the kit. Primers and amplicon lengths were listed in Table 1. Amplifications were performed using a Mastercycler Gradient-Eppendorf® thermal cycler. Thermocycling conditions were presented in Table 2. PCR products were verified by 2% agarose gel electrophoresis and ethidium bromide staining. 

**Table 1:** Primers of the TUBA1A, TUBB2B, and TUBB3 genes.

Gene	Exon/Part	Forward primer(5’->3’)	Reverse primer(5’->3’)	Amplicon length(bp)
TUBA1A	1	TTCTAACCCCAGTCCCCTTT	CCTCGCCCAGAGAGCTTAC	507
	2	TGTATCGTGCTGGGGATATG	AGAACATGATGGGGGAGGA	509
	3	GTGCTGGGACAGGAGGTCT	AAATAACAGTTCAATTCTGTGTTTGA	361
	4a	TTTTGGGGTTTTTAAAATTCAG	GACGCTCAATATCGAGGTTTCT	487
	4b	CCCTGGAGCACTCTGATT	AAATGGACAGCTTGGGTCTG	949
	4c	GACCAAGCGTACCATCCAGT	AAATGGACAGCTTGGGTCTG	522
TUBB2B	1	CACCCTCCTTGCATAAAAGC	GCGAAAGTCACCTCCTAGCC	375
	2	ATTTCATTGTGAGCCTTGGC	GCAAGGGAAAGGGGAGAAG	238
	3	CTTTGTTTGGGGCAACAT	CTGGCAATCACACCTCTTCA	288
	4a	TGAGGGGTTTGAGGTAAGGT	ACCTCCTTCATGGACATGCG	768
	4b	ACCCAGCAGATGTTCGACTC	GCCAGGTTATCGTCCCG	59
TUBB3	3	GGCTCTTAGGATGTGAGCAGG	GGTCTGCCATCAGAGCTTGG	259
	4a	AAGACAGAACAGGCATGGGG	GGGATCCACTCCACGAAGTA	877
	4b	GTTCGATGCCAAGAACATGA	GGGTTTAGACACTGCTGGCT	540

**Table 2:** Thermocycling conditions of TUBA1A, TUBB2B, and TUBB3 genes.

Stage	Temperature	Time	Number of cycles
Initial denaturation	94 °C	15 min	1
Denaturation	94 °C	30 s	35
Annealing	60 °C	45 s	
Extension	72 °C	45 s	
Final extension	72 °C	7 min	1
For TUBA1A part 4b and TUBB3 part 4a			
Initial denaturation	95 °C	15 min	1
Denaturation	95 °C	20 sec	35
Annealing	60 °C	40 sec	
Extension	72 °C	1 min	
Final extension	72 °C	7 min	1

BigDye Terminator v3.1 Cycle Sequencing Kit (Applied Biosystems) was used for the second PCR step. Second PCR conditions were as follows: Initial denaturation step at 96 °C for 1 min, followed by 25 cycles including denaturation at 96 °C for 10 s, annealing at 50 °C for 5 s, and extension at 60 °C for 4 min. Then, Zymo Research DNA Sequencing Clean-up Kit™ was used to purify the PCR products. Protocols were performed according to the standards provided with the kits. Purified PCR products were analyzed using ABI 3130 capillary electrophoresis system (Applied Biosystems, Thermo Fisher Scientific). 

CLC Genomics Workbench 3.6.5 sequencing software (Qiagen) was used for analysis. ENST00000301071.11, ENST00000259818.7 and ENST00000554444.5 transcripts in the ENSEMBLE database www.ensembl.org; GRCh38.p7, GCA_000001405.22 were used as reference sequences. All variations were interpreted using mutation and single nucleotide polymorphism (SNP) databases and in silico programs (Human Genome Mutation Database http://www.hgmd.cf.ac.uk, ClinVar https://www.ncbi.nlm.nih.gov/clinvar/, National Center for Biotechnology Information/SNP http://www.ncbi.nlm.nih.gov/snp, Ensembl, PolyPhen 2 http://genetics.bwh.harvard.edu/pph2/, and Mutation Taster http://www.mutationtaster.org/). Each variation was confirmed by bidirectional sequencing. All variants were classified according to 2015 ACMG standards and guidelines for the interpretation of sequence variants, and they were described according to the nomenclature recommended by the Human Genomic Variation Society. In case of novel genetic variations, the patients’ parents were also taken into consideration.

## 3. Results

A total of 47 patients (25 females, 22 males) with simple or complex MCD, as determined by radiological studies and with no diagnosis of recognizable genetic syndromes, were analyzed for *TUBA1A, TUBB2B,* and *TUBB3* mutations. The mean age of patients was 6.3 years (range 1 month and 17 years). Prenatal history was remarkable in 7 (14.9%) patients. Consanguineous marriage was reported in 8 (17%) families.

In the present study, the most common clinical findings were intellectual disability (83%, n = 39) and epilepsy (55.3%, n = 26) followed by microcephaly (29.8%, n = 14), macrocephaly (14.9%, n = 7), hypotonia (14.9%, n = 7), strabismus (14.9%, n = 7), and abnormal cerebellar findings (10.6%, n = 5). EEG abnormalities were reported in 24 (51.1%) patients. The most seen seizure type was generalized tonic clonic seizures (48.1%, n = 13). Clinical and cranial MRI findings were presented in Table 3.

Simple MCD was reported in 19 (40.5%) patients. Focal cortical dysplasia (n = 6, 12.8%) was the most common simple MCD subtype. Five patients (10.6%) had heterotopia, three patients (6.4%) had schizencephaly, three patients (6.4%) had polymicrogyria, and two patients (4.2%) had lissencephaly. Twenty-eight patients (59.5%) were classified as complex MCD. Lissencephaly was the most common coexisting cortical malformation, whereas corpus callosum anomalies were the most common coexisting extracortical neurodevelopmental abnormalities. The classification of MR findings was shown in Table 4. 

Sequencing analysis demonstrated no pathogenic alterations on exons and/or exon-intron junctions. Variations were detected in these three genes summarized in Table 5. On *TUBA1A* gene, linkage disequilibrium for rs1056875 A>G and rs697624 G>C polymorphisms was found in 28 patients. For *TUBA1A* gene rs1056875 A>G SNP, 19 patients (40.4%) had AA genotype, 23 patients (49%) had AG genotype, and 5 patients (10.6%) had GG genotype. In our patient group, the frequency of A allele was 65% (n = 61), and the frequency of G allele was 35% (n = 33). For *TUBA1A* gene rs697624 G>C SNP, 19 patients (40.4%) had GG genotype, 23 patients (49%) had GC genotype, and 5 patients (10.6%) had CC genotype. In our patient group, the frequency of G allele was 65% (n = 61), and the frequency of C allele was 35% (n = 33).

On *TUBA1A* gene, rs199717430 C>T (11:c.226+10 C>T) was detected on a single allele in a 12-year-old male patient with linear heterotopia. The patient’s parents were also analyzed due to lack of sufficient population studies. The results showed that the patient’s healthy father with normal brain MRI also carried the same variation in heterozygous state.

**Table 3:** Clinical and cranial MRI findings

PatientNo	Sex	Age	PH	HC	ID/MDD	Epilepsy/ST	EEG Abn.	NE	OE	CM	MR imaging
1	M	8 years	-	N	+	-	-	N	N	-	FCD, PFA
2	F	12 years	-	Mac.	+	+/GTC	+	N	N	-	FCD, Het.
3	M	9 months	-	N	+	-	-	DTR↑, hypo.	N	-	Lis., CCA
4	F	6 years	-	N	+	-	-	N	N	-	FCD
5	F	9 years	-	N	+	-	-	N	N	-	FCD
6	F	7 months	+	N	N	-	-	N	N	-	FCD
7	M	4 years	-	N	+	+/Focal	+	N	Strab.	-	FCD, PFA
8	F	2 years	-	Mic.	+	-	-	N	N	-	Het.
9	M	8 years	-	N	+	+/SGTC	+	N	N	-	FCD, CCA
10	F	10 years	-	Mic.	+	+/GTC	+	Spas.	N	-	FCD, CCA
11	F	11 years	-	N	N	-	-	N	N	+	Lis., CCA
12	F	12 years	-	N	N	+/Myoclonic	-	N	Ptosis	+	Het.
13	M	3 months	-	N	NA	-	-	Hypo.	N	-	Lis., PMG
14	F	7 years	-	Mic.	+	+/GTC	+	DTR↑,PR, Spas.	N	-	Lis., FCD
15	F	5 years	+	Mac.	+	+/GTC	+	DTR↑	N	-	FCD, PFA
16	M	15 years	-	Mac.	+	+/GTC	+	N	N	+	FCD
17	M	8 years	-	Mac.	+	+/Absans	+	N	N	-	FCD, Het.
18	F	8 years	-	N	+	+/GTC	-	DTR↑, Spas.	N	-	Sch.
19	M	13 years	-	N	N	+/GTC	+	Hypo.	N	-	Het., PFA
20	M	3 months	-	N	+	+/SGTC	+	PR	Nys.	-	PMG
21	M	13 years	-	Mic.	+	-	+	Spas.	N	-	Lis., PFA
22	M	17 years	-	N	+	+/GTC	+	N	Strab.	-	Lis.,Sch.
23	M	12 years	-	Mic.	+	+/Clonic	+	Hypo.	N	-	FCD
24	F	1 months	-	Mic.	NA	+/SGTC	+	DTR↑,PR, Spas.	N	-	Lis., PMG
25	F	11 years	+	N	N	-	-	N	N	-	FCD, CCA
26	M	6 years	-	N	+	-	-	DTR↑	N	-	Sch.
27	M	12 years	-	N	+	+/Focal	+	N	N	-	Het.
28	F	10 years	-	Mac.	+	-	-	N	N	-	Het.
29	F	4 years	+	Mic.	+	-	-	N	N	-	Lis.
30	F	3 years	-	N	+	-	-	Hypo.	N	+	Sch.
31	F	4 years	+	Mic.	+	+/Focal	+	DTR↑,PR, Spas.	N	-	PMG
32	F	7 years	-	Mic.	+	+/GTC	+	Spas.	N	-	Het., CCA
33	M	1 months	+	Mac.	NA	-	-	N	N	-	Het., CCA, PFA
34	F	13 years	-	N	+	-	-	N	N	+	FCD, PFA
35	M	3 years	+	Mac.	+	+/SGTC	+	N	N	-	Het.
36	M	3 years	-	Mic.	+	-	-	N	N	+	Sch., CCA
37	F	9 years	-	N	+	+/Focal	+	DTR↑,PR, Spas.	Strab.	-	Het., CCA, PFA
38	F	2 years	-	N	+	-	-	N	Strab.	-	Het., PFA
39	M	6 years	-	N	+	-	-	N	N	-	Het., CCA
40	F	1 years	-	Mic.	+	+/GTC	+	Hypo.	Strab.	-	Het., CCA, Lis.
41	F	1 years	-	N	+	-	-	DTR↑,PR, Spas.	Strab.	-	FCD
42	M	3 years	-	Mic.	+	+/GTC	+	N	N	+	Lis.
43	M	8 years	-	N	+	+/SGTC	+	DTR↑	N	-	PMG
44	F	5 months	-	Mic.	+	+/Tonic	+	Hypo.	N	+	FCD, CCA
45	F	9 years	-	N	+	-	-	N	N	-	FCD, PFA
46	M	17 years	-	Mic.	+	+/GTC	+	Spas.	Strab.	-	FCD, CCA
47	M	5 years	-	N	+	+/GTC	-	N	N	-	Het., PFA

F: female, M: male, PH: remarkable prenatal history, HC: head circumference, Mic: microcephaly, Mac: macrocephaly, N: normal, ID: intellectual disability, MDD: motor developmental delay, ST: seizure type, GTC: generalized tonic clonic, SGTC: secondary generalized tonic clonic, NE: neurologic examination, Hypo: hypotonia, Spas: spasticity, PR: pathologic reflex, OE: ophtalmologic examination, Strab: strabismus, Nys: nystagmus, CM: consanguineous marriage, FCD: focal cortical dysplasia, CCA: corpus callosum anomaly, PFA: posterior fossa anomaly, Het: heterotopia, Lis: lissencephaly, Sch: schizencephaly, PMG: polymicrogyri

**Table 4:** Classification of MR images.

Simple MCD		Complex MCD	
FCD	6	FCD+CCA	5
Heterotopia	5	FCD+PFA	5
Lissencephaly	2	FCD+Heterotopia	2
Schizencephaly	3	FCD+ Lissencephaly	1
Polymicrogyria	3	Heterotopia+CCA	2
		Heterotopia+PFA	3
		Heterotopia+PFA+CCA	2
		Lissencephaly+CCA	2
		Lissencephaly+PFA	1
		Lissencephaly+CCA+ Heterotopia	1
		Schizencephaly+CCA	1
		Schizencephaly+Lissencephaly	1
		Polymicrogyria+Lissencephaly	2
Total	19		28

MCD: malformations of cortical development, FCD: focal cortical dysplasia, CCA: corpus callosum anomaly, PFA: posterior fossa anomaly

**Table 5:** Variations were detected in three genes.

Gene	TUBA1A											
Variation	rs1056875 A>G(11:c.288 A>G)(p.K96K)				rs697624 G>C(11:c.453 G>C)(p.S151S)				rs199717430 C>T (11:c.226+10 C>T)			
Genotype	AA	AG		GG	GG	GC		CC	CC	CT		TT
%	40.4	49		10.6	40.4	49		10.6	98	2		0
n	19	23		5	19	23		5	46	1		0
Gene	TUBB2B											
Variation	rs199547345 C>T (7:c.718 C>T) (p.L240L)						rs200624566 C>T (7:c.609 C>T) (p.D203D)					
Genotype	CC		CT		TT		CC		CT		TT	
%	80.9		19.1		0		98		2		0	
n	38		9		0		46		1		0	
Gene	TUBB3											
Variation	rs147245174 C>T(5:c.486 C>T)(p.S162S)				rs141064323 G>A(5:c.999 G>A)(p.E333E)				rs61743676 C>T (5:c.450 C>T) (p.Y150Y)			
Genotype	CC	CT		TT	GG	GA		AA	CC	CT		TT
%	93.6	6.4		0	93.6	6.4		0	98	2		0
n	44	3		0	44	3		0	46	1		0

## 4. Discussion

Tubulin mutations are seen in 1–13% of all MCDs [7]. In this study, we investigated *TUBA1A* , *TUBB2B* , *TUBB3* mutations on 47 simple and complex MCD patients. We did not find any pathogenic variations, whereas some polymorphisms were more frequently detected than reported before.

Mutations on tubulin genes cause tubulinopathies, which include varying clinical findings (cognitive and motor impairments, refractory epilepsy, congenital microcephaly) and a broad spectrum of brain malformations (microlissencephaly, classical lissencephaly, polymicrogyria, schizencephaly, corpus callosum agenesia or hypoplasia, dysmorphic basal ganglia, brain stem and cerebellar hypoplasia, ventriculomegaly, and vermis hypoplasia) [7,9].

In our study, the most common clinical findings were intellectual disability (83%, n = 39) and epilepsy (55.3%, n = 26) followed by microcephaly (29.8%, n = 14), macrocephaly (14.9%, n = 7), hypotonia (14.9%, n = 7), strabismus (14.9%, n = 7) and abnormal cerebellar findings (10.6%, n = 5). EEG abnormalities were reported in 24 (51.1%) patients. The most seen seizure type was generalized tonic clonic seizures (48.1%, n = 13).

The most common type of MCD in present study was focal cortical dysplasia followed by heterotopia, lissencephaly, schizencephaly, and polymicrogyria. Twenty-eight patients (59.5%) were classified as complex MCD. Lissencephaly was the most common coexisting cortical malformation, while corpus callosum anomalies were the most common coexisting extracortical neurodevelopmental abnormalities. 

DNA sequencing was used to screen *TUBA1A, TUBB2B,* and *TUBB3* mutations. None of the patients had any genomic alterations on exon sequences and exon-intron junctions, which could disrupt protein function (mutation). We found linkage disequilibrium for rs1056875 A>G and rs697624 G>C polymorphisms on *TUBA1A* . Similarly, Rosendahl et al. [10] explored *TUBA1A* mutations in 46 patients with lissencephaly, and reported rs1056875 A>G and rs697624 G>C single nucleotide polymorphisms (SNPs) showing linkage disequilibrium. 

rs199717430 C>T SNP (11:c.226+10 C>T), which is located on intron 2 splice+10 of *TUBA1A* and creates a missense intronic variant was detected on a single allele in a 12-year-old male patient with linear heterotopia. Population studies have shown that the frequency of T allele is less than 0.1% in all populations. This variation is recorded as a “likely benign allele” in databases (ClinVar). Due to the lack of sufficient population studies, the patient’s parents were also analyzed. The patient’s father also had the same variation in a heterozygous state, and he had normal phenotype. Considering that i) the patient’s father has a normal phenotype, ii) the variation is located on the intron, and iii) autosomal dominant inheritance of *TUBA1A* mutations, this variation is not likely to be responsible for the observed phenotype. 

In conclusion, the patient group in the present study represents a significant patient population. We did not find any pathogenic variation in any of three genes, and it is likely that small sample size and the high number of genes responsible for MCDs are main reasons for this observation. Furthermore, the rate of consanguineous marriages in our study (17%) was higher than the rates of developed countries (<5%) [11]. Consanguineous marriages are associated with an increased risk for autosomal recessive diseases; therefore, the high risk of having an autosomal recessive disease is another reason to consider. We think that multicenter studies with higher patient numbers and also gene panels including other MCD-related genes are required to determine underlying etiological factors of MCD patients.
